# Should disease-modifying therapy be discontinued in older patients with stable MS after long-term clinical and radiological stability? Commentary

**DOI:** 10.1177/13524585251344786

**Published:** 2025-05-26

**Authors:** Anna Karin Hedström

**Affiliations:** Department of Clinical Neuroscience, Karolinska Institutet, Stockholm, Sweden

The question of whether disease-modifying therapies (DMTs) should be discontinued in older patients with multiple sclerosis (MS) who have achieved long-term stability is both clinically important and increasingly relevant. As more patients age while on DMT, clinicians are often faced with balancing potential benefits against evolving risks.

In this issue of MS Journal, Foong and Butzkueven argue against routine DMT discontinuation, pointing to evidence that disease reactivation can still occur in older individuals, even after extended periods of clinical and radiological stability. They emphasize the difficulty in predicting individual outcomes and the potential for irreversible disability if relapses or MRI activity return following cessation. In contrast, Androdias and Stankoff underscore that with advancing age and disease duration, the inflammatory component of MS tends to wane, reducing the primary target of most DMTs. Combined with the growing risks of long-term immunosuppression, such as infections and malignancies, they argue that treatment discontinuation in selected cases may be appropriate and aligned with long-term safety.

Both perspectives are well supported. In the DISCOMS trial, which enrolled patients aged 55 and older with no recent clinical relapses or MRI activity, those who discontinued therapy had a modest risk of recurrent disease activity.^1^ Most new activity was subclinical, and an extension study showed no relapses in either group, with low rates of new MRI lesions.^2^ These findings are consistent with broader observational data showing that relapses become increasingly rare with age.^3^ However, as Foong and Butzkueven note, clinically silent radiological activity may not be benign, and its long-term consequences remain unclear.

Importantly, both sides note that the risk of reactivation varies by DMT type. Discontinuing DMTs that alter lymphocyte trafficking carries a higher risk of rebound disease activity,^4^ suggesting that a drug-specific approach is needed rather than one-size-fits-all recommendations.

These findings suggest that while discontinuation may not be appropriate for all, it may be a safe and viable option for carefully selected patients, particularly those on platform therapies. Longer-term prospective studies and registry data are needed to better stratify risks and guide clinical decisions. Further research, including the identification and validation of biomarkers to support discontinuation strategies, is also urgently needed.

Rather than complicating clinical practice, this debate affirms the value of individualized decision-making. Foong and Butzkueven rightly caution against a blanket discontinuation policy in the absence of reliable biomarkers to predict reactivation. Meanwhile, Androdias and Stankoff argue that for stable patients on lower risk therapies, discontinuation may be a reasonable and patient-centered goal, emphasizing as well that the healthcare costs and treatment burden of continued therapy are not negligible considerations.

Until more guidance is available, the most sensible path lies not in strict adherence to either continuation or discontinuation, but in a flexible, individual approach, accounting for age, disease course, treatment type, duration of stability, comorbidities, and patient preference.

## References

[1] Corboy JR Fox JR Kister I , et al. Risk of new disease activity in patients with multiple sclerosis who continue or discontinue disease-modifying therapies (DISCOMS): A multicentre, randomized, single-blind, phase 4, non-inferiority trial. Lancet Neurol 2023; 22: 568–577. 37353277 10.1016/S1474-4422(23)00154-0

[2] Corboy JR Fox RJ Cutter G , et al. Discontinuation of disease-modifying therapies in MS: The DISCOMS extension trial. Mult Scler 2025; 31(2): 159–173. 39834328 10.1177/13524585241303489

[3] Tremlett H Zhao Y Joseph J , et al. Relapses in multiple sclerosis are age- and time-dependent. J Neurol Neurosurg Psychiatry 2008; 79(12): 1368–1374. 18535026 10.1136/jnnp.2008.145805

[4] Jouvenot G Courbon G Lefort M , et al. High-efficacy therapy discontinuation vs continuation in patients 50 years and older with nonactive MS. JAMA Neurology 2024; 81: 490–498. 38526462 10.1001/jamaneurol.2024.0395 PMC10964164

